# Hypertonicity during a rapid rise in D-glucose mediates first-phase insulin secretion

**DOI:** 10.3389/fendo.2024.1395028

**Published:** 2024-06-26

**Authors:** Varun Kamat, Ian R. Sweet

**Affiliations:** University of Washington Medicine Diabetes Institute, University of Washington, Seattle, WA, United States

**Keywords:** insulin secretion, cAMP, L-type calcium channel, biphasic insulin secretion, islet, hypertonicity, osmotic pressure, first phase

## Abstract

**Introduction:**

Biphasic insulin secretion is an intrinsic characteristic of the pancreatic islet and has clinical relevance due to the loss of first-phase in patients with Type 2 diabetes. As it has long been shown that first-phase insulin secretion only occurs in response to rapid changes in glucose, we tested the hypothesis that islet response to an increase in glucose is a combination of metabolism plus an osmotic effect where hypertonicity is driving first-phase insulin secretion.

**Methods:**

Experiments were performed using perifusion analysis of rat, mouse, and human islets. Insulin secretion rate (ISR) and other parameters associated with its regulation were measured in response to combinations of D-glucose and membrane-impermeable carbohydrates (L-glucose or mannitol) designed to dissect the effect of hypertonicity from that of glucose metabolism.

**Results:**

Remarkably, the appearance of first-phase responses was wholly dependent on changes in tonicity: no first-phase in NAD(P)H, cytosolic calcium, cAMP secretion rate (cAMP SR), or ISR was observed when increased D-glucose concentration was counterbalanced by decreases in membrane-impermeable carbohydrates. When D-glucose was greater than 8 mM, rapid increases in L-glucose without any change in D-glucose resulted in first-phase responses in all measured parameters that were kinetically similar to D-glucose. First-phase ISR was completely abolished by H89 (a non-specific inhibitor of protein kinases) without affecting first-phase calcium response. Defining first-phase ISR as the difference between glucose-stimulated ISR with and without a change in hypertonicity, the peak of first-phase ISR occurred after second-phase ISR had reached steady state, consistent with the well-established glucose-dependency of mechanisms that potentiate glucose-stimulated ISR.

**Discussion:**

The data collected in this study suggests a new model of glucose-stimulated biphasic ISR where first-phase ISR derives from (and after) a transitory amplification of second-phase ISR and driven by hypertonicity-induced rise in H89-inhibitable kinases likely driven by first-phase responses in cAMP, calcium, or a combination of both.

## Introduction

It well known that step increments of glucose concentration trigger biphasic insulin secretory response that consists of an initial burst of insulin release in the first 3–10 minutes termed first-phase followed by a sustained second phase. This response is observed during intravenous glucose tolerance tests (IVGTT) in humans (1, 2), in perfused mammalian pancreas (3), and in perifused isolated pancreatic islets (4), observations that were made over 50 years ago. The mechanisms mediating the complex insulin secretory dynamics have both fundamental and clinical significance, as the first-phase is absent (or attenuated) in IVGTT’s performed on patients with Type 2 diabetes mellitus (5–7). Although the fundamental elements mediating glucose-stimulated ISR have been identified including glucose sensing by glucokinase, depolarization of the cell membrane by closure of K_ATP_ channels, and the resultant opening of L-type calcium channels, often referred to as the “consensus model” (8), there is still much disagreement on the biochemical and biophysical processes underlying biphasic release of insulin. The topic of how the biphasic response arises has been presented in multiple and thoughtful reviews (8–13) and many mechanisms/signaling molecules mediating the phasic nature of glucose-stimulated ISR have been proposed. These include calcium (8, 14, 15), a two pool compartmental model of insulin granules (16) (one of which would correspond to a “readily releasable pool” (17, 18) involving cytoskeleton components acting to separate out ready releasable granules from internal stores of granules (19, 20)), direct action of K_ATP_ channels on SNARE proteins (21), cell-to-cell heterogeneity (22), osmotic pressure (23), GABA (24), PI3 kinase (25), ATP/ADP (26), and phospholipase A (27). The interaction between paracrine/juxtacrine signals from other islet cell types has also been considered as factors impacting the waveform of secretion, most notably glucagon and somatostatin (28), the EphA receptor/ephrin-A system (29, 30), and cAMP that is a potent stimulator of secretion in both alpha and beta cells (31). The list of possibilities is extensive and indeed, there is also little consensus on whether mechanisms that mediate the two phases are independent, such as that supported by data from beta-cell specific KO of the insulin receptor (32), or the view that second phase is simply multiple iterations of the same mechanism (8). Thus, despite the importance of understanding glucose regulation of ISR, the underlying factors giving rise to this behavior are not established.

It has long been recognized that generating first-phase ISR during perifusions is dependent on the rapidity of the change in glucose (33). Fast flow rates used in perifusion studies which can facilitate rapid changes in glucose concentrations during stimulation produce biphasic insulin secretion, whereas slow flow rates (such as those used when measuring O_2_ consumption) generate only second-phase response when stimulating with glucose (34–36). Likewise, in *in vivo* studies, IVGTTs produce first-phase insulin secretion, whereas response of insulin secretion to a meal is not biphasic (37). Thus, it is well established that the rate of change of glucose governs the generation of first-phase insulin secretion. Accordingly, studies conducted by Best and Yates were carried out to investigate the effect of osmotic changes on ion channel regulation (23). They found that very high levels of hypertonicity (i.e., 100 mM sucrose) can induce insulin secretion. possibly via Non-Selective Cation Channels and suggested that this mechanism “could underlie the biphasic effect on insulin release following exposure to hypertonic media” (23). Nonetheless, use of such high levels of sucrose is not immediately pertinent to changes in glucose under typical experimental conditions. A paper reported by the same group described a non-metabolizable glucose analog, 16 mM 3-O-methylglucose, had a small effect on cell volume but no effect on membrane potential and concluded that glucose must be metabolized to elicit an osmotic response (38). Based on reports that glucose leads to swelling of islet cells and hypotonic conditions (38), there have been several studies on the effects of osmotic pressure on islet function mediated by various stretch activated cation channels (39–41). These include piezo1, Tentonin 3/TMEM150, TRPV2 and LRRC8/VRAC, all of which have been reported to support ISR in response to glucose-induced hypotonicity (42–45).

Since slow increases in glucose fail to elicit first-phase insulin secretion and a rapid increase in D-glucose will at least initially cause an increase in tonicity rather than a decrease, it made sense to consider whether a rapid rise in glucose eliciting first-phase ISR would be mediated by hypertonicity. Specifically, we postulated that the biphasic ISR waveform was the result of two separate driving forces: 1. an abrupt increase in hypertonicity that elicits first-phase ISR; and 2. phosphorylation and further metabolism of glucose that generates second-phase ISR by canonical well-established mechanisms.

We therefore endeavored to test this postulate by experimentally separating out these two driving forces while measuring insulin secretion rate (ISR), NAD(P)H (reflecting changes in glucose metabolism) and key signals mediating insulin secretion (calcium and cAMP) using a custom—made islet perifusion system (46–48). To do the bulk of the work, rat islets were used as mouse islets are particularly electrically active and display greater oscillatory characteristics, which would complicate the resolution of first-phase responses. To achieve the increase in osmolarity without an increase glucose metabolism, we utilized L-glucose, that has been shown in islets (49, 50) and liver (51) to be membrane-impermeable due to the stereospecificity of glucose transporters. To achieve the increase in D-glucose metabolism without a change in osmolarity, islets were perifused with a solution containing L-glucose (or another membrane impermeable carbohydrate mannitol) which was then replaced with D-glucose so that the decrease in the L-glucose concentration is exactly matched to the increase in D-glucose. The osmotic coefficients for the test compounds are essentially identical (1.0 for mannitol, 1.01 for L- and D-glucose) (52). When D-glucose is abruptly increased, an increase in both osmolarity and glucose metabolism occurs. Performing these three protocols we measured ISR, NAD(P)H, calcium, and cAMP to reveal effects mediated by hypertonicity. This direct experimental approach revealed that all measured parameters respond biphasically to a rise in L- or D-glucose concentration, where the first-phase components were wholly related to hypertonicity occurring during a rapid rise in glucose, and where first-phase ISR depended on a process involving H89-sensitive kinase(s).

## Results

### First-phase ISR requires hypertonicity

**Figure 1:**
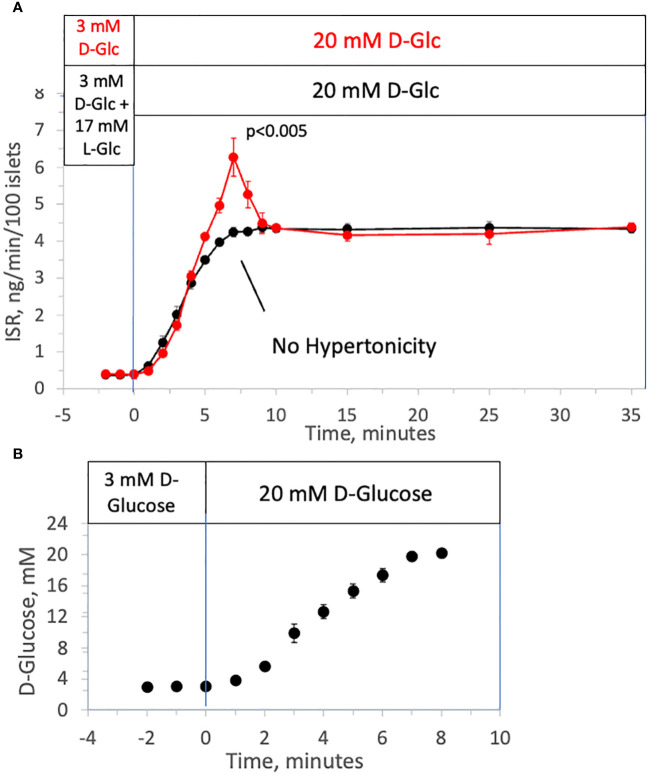
Effect of hypertonicity on insulin secretion from rat islets during rapid increases to 20 mM D-glucose. **(A)** ISR in response to a rapid increase in the concentration of D-Glucose with and without changes in tonicity. Rat islets (50/channel) were perifused at 3 mM D-glucose with or without 17 mM L-glucose for 90 minutes, after which the concentration of D-glucose was increased to 20 mM glucose without L-glucose. Insulin was measured in outflow fractions collected in 1-minute intervals. Each data point is an average +/- SE of 5 perifusions. Statistical significance shown in the graphs was calculated using t-tests comparing the peak of ISR following an increase in D-glucose with and without prior exposure to L-glucose. **(B)** D-Glucose was measured by colorimetric assay (that was unaffected by the presence of L-glucose) in outflow fractions collected from two minutes prior to the change to 20 mM glucose to 8 minutes following the change. Each data point is an average +/- SE of 4 perifusions. Flow rate was 250 mL/min in each 140 mL chamber.

Using a custom-made multi-channel perifusion system, we measured ISR from 50 isolated rat islets/channel (flow rate = 250 μL/min) (53) in response to a rapid increase in D-glucose with (control) or without a change in tonicity. Control islets in half of the chambers were first perfused with 3 mM D-glucose as a non-stimulatory baseline (for 90 minutes), which was then rapidly increased to 20 mM. The rapid change in D-glucose elicited the expected first-phase ISR (from 5 to 10 minutes, **Figure 1A**) followed by a sustained second phase. D-glucose was measured in the outflow samples and glucose increased from 3 to 20 mM in approximately 8 minutes corresponding to changes in osmolarity from 314 to 331 mOsm (**Figure 1B**); the increase next to the islets in the perifusion chamber although not measured would be faster than this. To measure the effect of glucose metabolism in the absence of a change in osmolarity, during the non-stimulatory baseline period, islets in the other chambers were perifused for 90 minutes with KRB containing 3 mM D-glucose and 17 mM L-glucose (a membrane-impermeable form of D-glucose). Subsequently, the 17 mM L-glucose was washed out at the same time D-glucose was increased from 3 to 20 mM D-glucose thereby maintaining iso-osmolar conditions. D-glucose in the outflow increased similarly to control channels that did not contain the L-glucose. Whereas the second phase ISR induced by D-glucose was unaffected, the first-phase was completely absent (**Figure 1A**) indicating that first-phase ISR depends on inducing changes in osmolarity. Notably, the onset of D-glucose stimulated ISR in the presence and absence of a change in L-glucose were superimposable. It should be noted that although a gradual increase in second-phase ISR is often observed (11), ISR responses to D-glucose in our study consistently reached a steady state within 5 minutes following the first-phase secretory period, and subsequently remained very stable for at least 30 minutes. It is known that the conditions islets are subjected to prior to exposing the islets to increased glucose greatly impact the waveform of biphasic insulin secretion (54). Therefore, it is likely that being pre-incubated for an atypically long period of 2 hours in KRB containing 3 mM glucose decreased the time it took for ISR to reach steady state.

### Rapid change in tonicity triggers first-phase ISR in the absence of a change in prevailing D-glucose level

**Figure 2:**
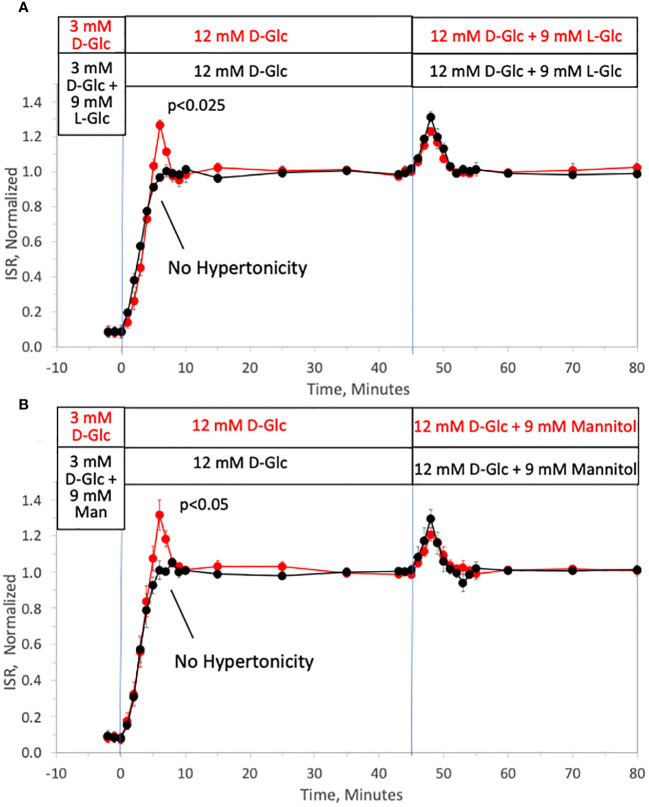
Dissection of osmotic vs metabolic components of biphasic insulin secretion in response to 12 mM D-glucose. Rat islets (50/channel) were perifused at 3 mM D-glucose with or without 9 mM L-glucose **(A)** or mannitol **(B)** for 90 minutes, after which the concentration of D-glucose was rapidly increased to 12 mM without any membrane impermeable compounds. Subsequently, L-glucose or mannitol was added in the absence of a change in D-glucose. Outflow fractions were collected in 1-minute intervals, and insulin was subsequently measured in the fractions at the indicated times. Each data point is an average +/- SE of 3 perifusions normalized to the steady-state value at 12 mM glucose calculated by dividing each value by the average ISR values between times of 25–45 minutes (**A**: 3.7 +/- 0.32 ng/min/100 islets for D-glucose alone and 3.4 +/- 0.26 ng/min/100 islets preceded by mannitol; and B: 3.6 +/- 0.26 ng/min/100 islets for D-glucose alone and 3.4 +/- 0.14 ng/min/100 islets preceded by L-glucose). Statistical significance shown in the graphs was calculated using t-tests comparing the peak of ISR following an increase in D-glucose with and without prior exposure to L-glucose **(A)** or mannitol **(B)**. Flow rate was 250 mL/min in each 140 mL chamber.

To investigate the ability of changes in tonicity to induce first-phase ISR in the absence of a change in D-glucose, the protocol used in **Figure 1** was repeated but with smaller D-glucose increments (from 3 to 12 mM glucose) and subsequently followed by addition of 9 mM of two different membrane-impermeable carbohydrates L-glucose (**Figure 2A**) or mannitol (**Figure 2B**) (corresponding to changes in osmolarity from 314 to 323 mOsm) once D-glucose had stabilized at 12 mM. As with increments to 20 mM D-glucose, peaks of the first-phase ISR in the controls occurred at 6–7 minutes following exposure to increased D-glucose. When changes in tonicity were counterbalanced by the concomitant washout of 9 mM L-glucose or mannitol, first-phase responses to 9 mM D-glucose increments were totally absent; second-phase ISR however was unaffected by counter-balancing tonicity with either impermeable carbohydrate. After second phase ISR was steady for 30 minutes, the membrane-impermeable compounds were added without a change in D-glucose. As was predicted, transient changes in ISR were observed that resembled the magnitude and waveform of first-phase ISR observed in response to D-glucose, except the peak occurred only 3 minutes following the initial exposure to the membrane-impermeable carbohydrates. Thus, only in response to changes in tonicity, irrespective of whether D-glucose is changing, does first-phase ISR waveform occur, and remarkably, the peak of the first-phase ISR occurs subsequent to the upslope in ISR elicited by the metabolic effects of D-glucose.

We also performed experiments where 9 mM L-glucose was rapidly removed in the presence of 12 mM D-glucose. Although it might have been expected to elicit a transient decrease in ISR, no changes in ISR were observed in response to the decrease in tonicity (data not shown). This is consistent with the fact that ISR in response to washout of D-glucose in perifusion systems has not revealed biphasic behavior or discontinuities in the decay of ISR (55, 56).

### Magnitude of osmotically triggered ISR depends on prevailing D-glucose concentration

**Figure 3:**
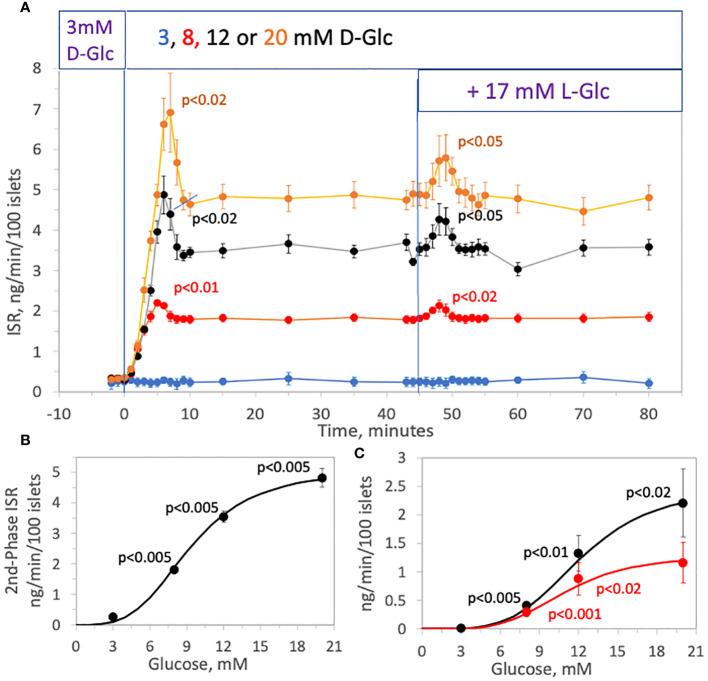
Effect of D-glucose concentration (3, 8, 12 or 20 mM) on the stimulation of ISR by hypertonicity. Rat islets (25/channel) were perifused at 3 mM D-glucose for 90 min, after which the concentration of D-glucose in the buffer was rapidly changed to either 3, 8, 12 or 20 mM, as indicated on the legend **(A)**. Subsequently, 17 mM L-glucose was added to all channels without a further change in D-glucose. Insulin was measured in selected outflow fractions that were collected in 1-min intervals. Each data point is an average +/- SE of 4 channels (8, 12, 20 mM D-glucose) or 3 channels (3 mM D-glucose). Statistical significance was calculated using a t-test that compared the peak of transient vs. the second phase ISR (steady state ISR was calculated as the average of the last 3 time points of the condition). Flow rate was 250 mL/min in each 140 mL chamber. **(B)** Steady-state (second phase) values of ISR for each D-glucose concentration was plotted and fit to a Hill plot (where V_max_ = 5.1 ng/min/100 islets, S _0.5_ = 9.3 mM, and Hill number = 3.5). Statistical significance was calculated using a t-test comparing ISR values at each D-glucose concentration with the ISR at the next highest glucose concentration. **(C)** Peak values for each transient (calculated as the difference in the peak minus the second phase ISR) following the change in D-glucose (black dots) and addition of L-glucose (red dots) of ISR for each D-glucose concentration was plotted and fit to a Hill plot (For first phase ISR following D-glucose: V_max_ = 2.5 ng/min/100 islets, S _0.5_ = 12.2 mM, and Hill number = 4.2; For first phase ISR following L-glucose: V_max_ = 1.3 ng/min/100 islets, S _0.5_ = 11.0 mM, and Hill number = 4.2). Statistical significance was calculated using a t-test comparing first phase ISR values at each D-glucose concentration with the first phase ISR at the next highest glucose concentration.

To test whether the effect of rapidly increasing tonicity on ISR was glucose-dependent, the effect of 17 mM L-glucose on ISR was tested in the presence of 3, 8, 12 and 20 mM, D-glucose (**Figure 3A**). The magnitude of both first- and second-phase ISR increased with increasing concentrations of D-glucose (**Figures 3B, C**). After second-phase ISR was steady for 30 minutes, 17 mM L-glucose was added without a change in D-glucose to each channel. At 3 mM D-glucose, adding L-glucose had no effect on ISR, and the transient ISR response to the same osmotic perturbation increased as a function of the prevailing glucose level. As with first-phase ISR elicited by D-glucose, amplitudes of the transient phase induced by the addition of L-glucose increased with D-glucose concentration (**Figure 3C**) and had a similar D-glucose dependency (S_0.5_ = 11.7 vs. 12.5 for first-phase of D-glucose-stimulated ISR).

**Figure 4:**
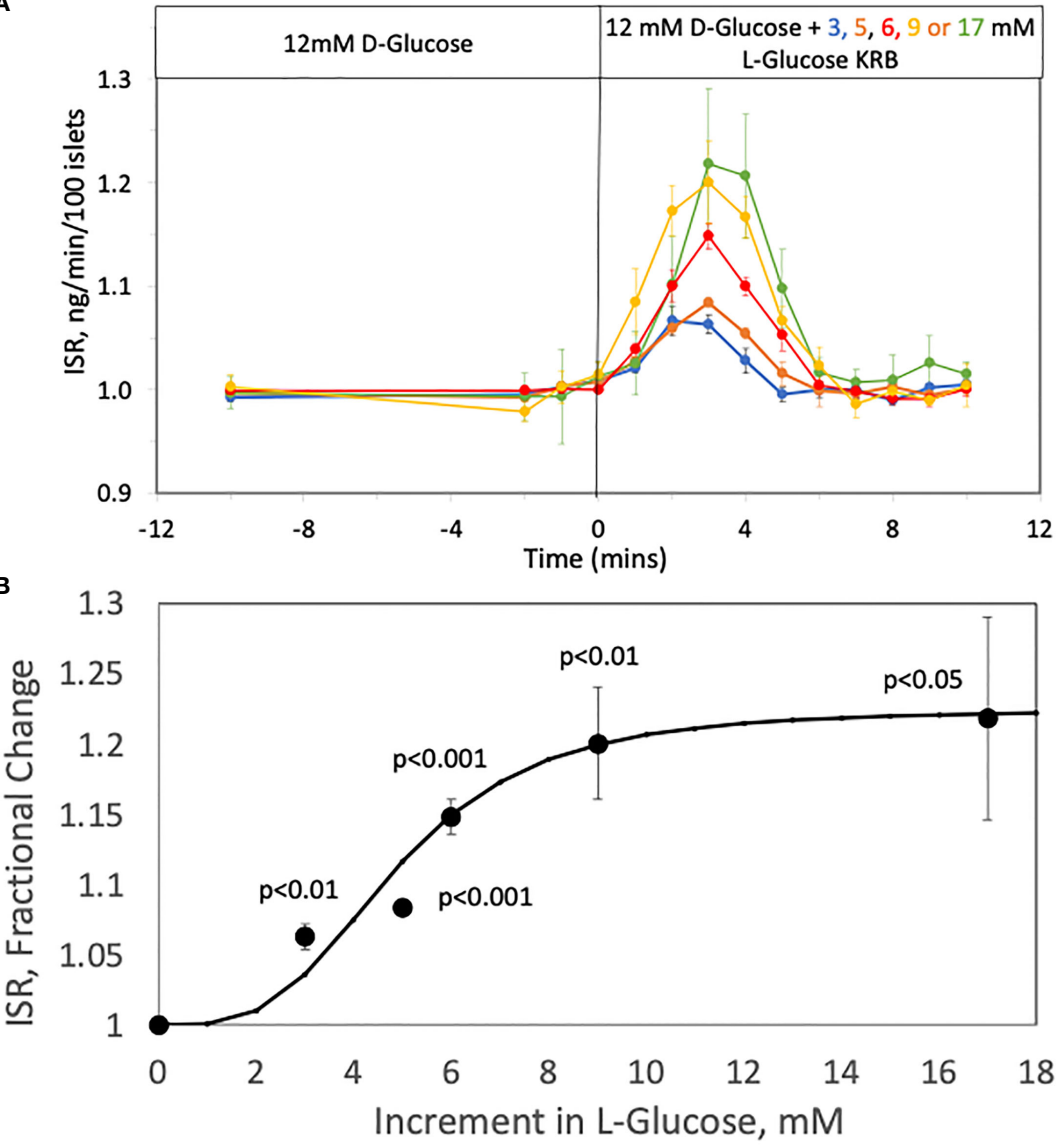
Concentration Dependency of the Effect of L-glucose on ISR at constant (12 mM) glucose. **(A)** Rat islets (25/channel) were perifused at 3 mM D-glucose for 90 min, after which the concentration of D-glucose in the buffer was rapidly changed to 12 mM. Subsequently, varying concentrations of L-glucose was added to channels without a further change in D-glucose. Insulin was measured in selected outflow fractions only in response to the L-glucose that were collected in 1-min intervals. Each data point is an average +/- SE of the following number of channels at the concentration of L-glucose: 4 (for 3, 9 and 17 mM), 6 (for 6 mM) and 5 (for 5 mM). Flow rate was 250 mL/min in each 140 mL chamber. **(B)** The peak values of ISR (normalized to the baseline) for each L-glucose concentration was plotted and fit to a Hill plot (where V_max_ = 0.23, S_0.5_ = 4.5 mM, and Hill number = 4.3). Statistical significance was calculated with a t-test by comparing ISR values at each glucose concentration with the ISR when no L-glucose was added.

To assess the relationship between increments in tonicity and magnitude of first-phase ISR we measured the concentration-dependency of ISR in response to varying increases in L-glucose in the presence of constant (12 mM) D-glucose. The protocol used in **Figure 2A** was repeated except that 3, 5, 6, 9, or 17 mM, L-glucose was added 45 minutes after increasing D-glucose to 12 mM. Insulin was measured in the outflow fractions before and after the addition of the L-glucose, revealing the L-glucose concentration-dependency of the responses of ISR (**Figure 4A**). The magnitude of the spikes become more difficult to resolve accurately below 5 mM L-glucose, but spikes were still significant in response to even 3 mM L-glucose which represents less than a 1% increase in osmolarity. The peak ISR was plotted as a function of L-glucose concentration (**Figure 4B**), where the fit of the data yielded a S_0.5_ of 4.5 mM.

### Cellular responses to osmotic triggers

As the well-accepted canonical model of glucose-stimulated ISR involves the processes of increased metabolism, and K_ATP_ channel-mediated depolarization of the cell membrane (57) leading to activation of LTCCs, we sought to connect the glucose dependency of first-phase insulin secretion with these steps. We therefore measured several critical control parameters in the canonical model including intracellular NAD(P)H (as a reflection of increased glucose metabolism) and cytosolic calcium concentration (by fluorescent imaging) reflecting activation of voltage-dependent calcium channels including LTCCs. In addition, we also measured cellular efflux rates of the cell signal cAMP, a factor known to potentiate glucose-stimulated ISR as well as increase in response to elevated glucose.

### Effect of tonicity on metabolic rate as reflected by NAD(P)H in rat islets

**Figure 5:**
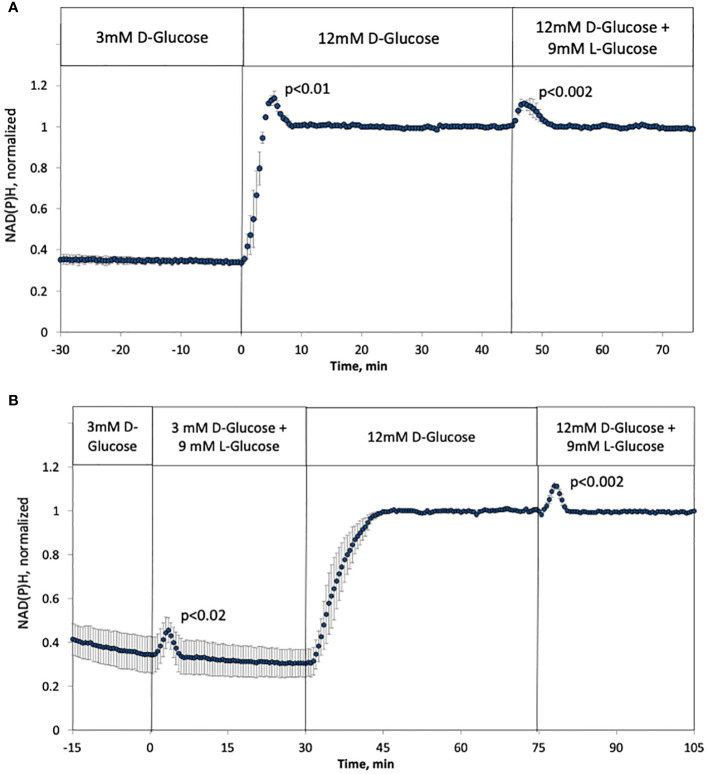
Effect of hypertonicity on intracellular NAD(P)H in rat islets with and without changes in D-glucose concentration (12 mM glucose). **(A, B)** The protocols carried out in **Figure 2A** were carried out while measuring NAD(P)H, except that the measurement of changes in response to L-glucose at 3 mM glucose was included **(B)**. Each data point is an average +/- SE of 3 separate imaging experiments, each on a single islet. Statistical significance was calculated using a t-test that compared the peak of the transient vs, the second phase NAD(P)H (steady state NAD(P)H calculated as the average of NAD(P)H data acquired from 10–20 minutes following the change in D- or L-glucose). Flow rate was 200 mL/min into each 100 mL chamber.

NAD(P)H increased in response to a rapid change from 3 to 12 mM glucose in a biphasic fashion with similar kinetics as with glucose-stimulated ISR (**Figure 5A**), which has also been seen by others (58). In the past, we have not seen first-phase responses with NAD(P)H (35, 48) as we used a slower flow rate (150 uL/min vs. 200 uL/min) that flowed into a larger perifusion chamber (250 uL vs 100 uL in the current setup). It is common to use 1 mL/min in assessment of human islets (55), so in this study we have carried out experiments near the minimal flow rate needed to observe biphasic responses. As with the response of ISR to L-glucose, a subsequent rapid change in L-glucose led to a transient increase in NAD(P)H in the face of constant concentration of D-glucose (**Figure 5A**), and no first-phase rise in NAD(P)H occurred in the absence of a change in tonicity that was prevented by replacing L-glucose with D-glucose (**Figure 5B**). Unexpectedly, addition of 9 mM L-glucose while islets were exposed to 3 mM D-glucose, increased NAD(P)H with kinetics and magnitude that were like that seen in the presence of 12 mM D-glucose. Thus, rapid changes in tonicity leads to transiently increased NAD(P)H values which was unaffected by prevailing levels of D-glucose.

### Effect of tonicity on first-phase intracellular calcium in rat islets

**Figure 6:**
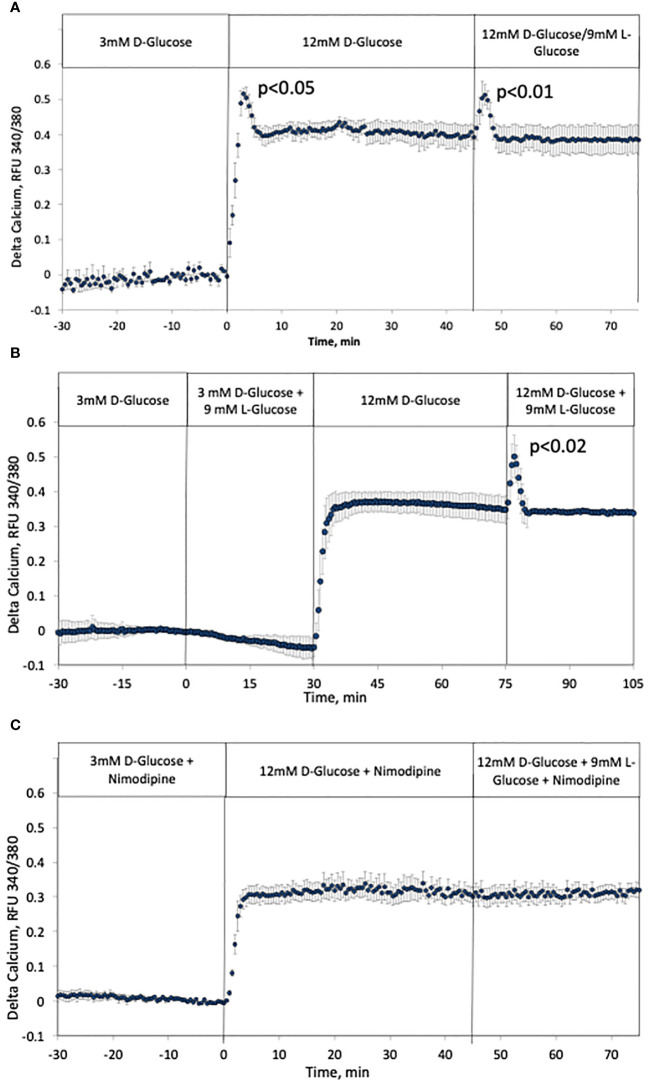
Effect of hypertonicity and contribution of LTCCs on intracellular calcium in rat islets. **(A, B)** The protocols carried out in **Figure 5**. were carried out while measuring intracellular calcium. Each data point is an average +/- SE of 3 separate imaging experiments. Statistical analysis was done as described in the Figure legend of **Figures 5A, B**. **(C)** The protocol in **(A)** was repeated in the presence of 5 uM nimodipine, a blocker of LTCCs. Each data point is an average +/- SE of 3 separate imaging experiments. Flow rates were 200 mL/min in each 100 mL chamber.

As has been previously recognized, the kinetics of intracellular calcium response to glucose is also biphasic (9, 59). Based on the NAD(P)H data, it was predicted that calcium would also be activated by rapid changes in osmolarity similarly to NAD(P)H and ISR. Recapitulating historical results, the response of calcium to increased D-glucose was biphasic and temporally similar to that of NAD(P)H and ISR, and the addition of L-glucose while maintaining constant D-glucose resulted in a transient response of calcium that mirrored first-phase rise (**Figure 6A**). Note that although it would be expected that calcium responses would precede ISR, the calcium measurements are made by imaging islet fluorescence, while ISR is measured in outflow fractions, precluding the resolution of the precise kinetic relationships between the two data sets. Closely mirroring the results with NAD(P)H and ISR, counterbalancing the rise in D-glucose by removal of L-glucose prevented the first-phase calcium response (**Figure 6B**). In contrast to NAD(P)H, adding L-glucose in the presence of 3 mM D-glucose did not affect calcium (**Figure 6B**). As ISR is preferentially sensitive to calcium entering beta cells via LTCCs (60) reflecting the operation of microdomains where LTCCs have been shown to be in close proximity to secretory granules (61, 62), we predicted that first-phase calcium responses would be mediated by LTCC activity. To test this prediction, the protocol in **Figure 6A** was carried out in the presence of nimodipine, a specific blocker of LTCCs (**Figure 6C**). Consistent with previous studies (35, 63), glucose-stimulated intracellular calcium in the presence of nimodipine was reduced, but the effect was smaller than expected (only 15%). Subsequently, in the presence of the LTCC blocker, no first-phase calcium response was observed either in response to D-glucose or L-glucose. Past studies where islets are exposed to nimodipine after their stimulation with increased D-glucose, observed an acute 40–50% decrease in calcium in response to nimodipine (35, 61, 63), indicating that most of the increase in calcium elicited by glucose is not mediated by LTCCs. The difference in the reduction in contributions of LTCC to glucose-stimulation of intracellular calcium could suggest that pre-incubation with nimodipine may lead to the upregulation of other calcium transporters. However, irrespective of the potential compensation by calcium channels independent of LTCCs, it appears first-phase calcium response to osmotic changes is entirely mediated by LTCCs.

### Effect of glucose-induced change in osmotic pressure on cAMP SR

**Figure 7:**
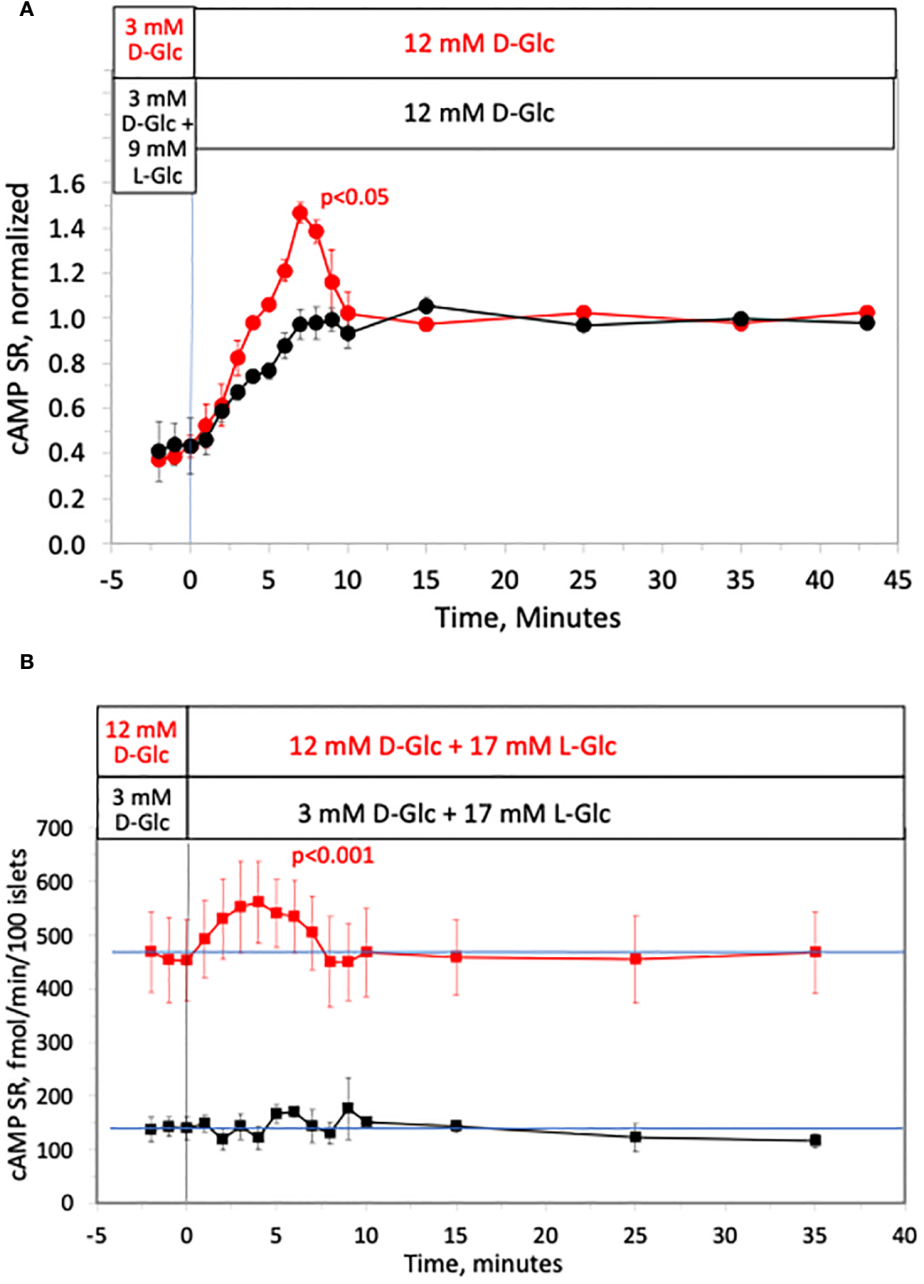
Effect of hypertonicity on cAMP response from rat islets. **(A)** Release of cAMP was assayed in the same fractions that were assayed for insulin in the first half of the protocol in **Figure 2A**. Each data point is an average +/- SE of 2 perifusions, normalized to the steady-state value at 12 mM glucose calculated by dividing each value by the average ISR values between times of 25–43 minutes (224 +/- 34 fmol/min/100 islets for D-glucose alone and 154 +/- 14 fmol/min/100 islets preceded by L-glucose). Statistical significance shown in the graphs was calculated using t-tests comparing the peak of ISR following an increase in D-glucose with and without prior exposure to L-glucose. **(B)** Release of cAMP assayed in the same fractions that were assayed for insulin in **Figure 3A**. Each data point is an average +/- SE of either 4 (at 12 mM D-Glc) or 3 channels (at 3 mM D-glucose). Statistical significance was calculated using a t-test that compared the peak of transient vs. the second phase cAMP (steady state cAMP calculated as the average of the last 3 time points of the condition). Flow rate was 250 mL/min in each 140 mL chamber.

The second messenger cAMP strongly potentiates glucose-stimulated ISR through the actions of PKA and also EPAC (64), and is also generated in response to increased glucose metabolism (65). As an alternative to measuring cAMP by fluorescent imaging (66), or content (67, 68), in order to resolve kinetics and magnitude of changes in cAMP we measured the rate of cAMP released into the perifusate by assaying the outflow fractions that were collected. As was the case for ISR, calcium and NAD(P)H, cAMP SR increased biphasically in response to a rapid increase in D-glucose, where first-phase was not observed when the osmotic change was masked by concomitant removal of L-glucose (**Figure 7A**). Here data was normalized to highlight the relative waveform of the response. Moreover, addition of L-glucose in the face of 12 mM D-glucose generated a first-phase response in cAMP SR (**Figure 7B**). As with calcium and ISR, the effect of L-glucose was dependent on the concentration of D-glucose and no effect was seen at 3 mM glucose (**Figure 7B**). Thus, it appears that although NAD(P)H increased in response to hypertonicity even at low glucose, downstream processes of cAMP and calcium require elevated glucose concentration to respond to changes in osmolarity.

### Inhibition of first-phase ISR with H89 without affecting first-phase calcium response

**Figure 8:**
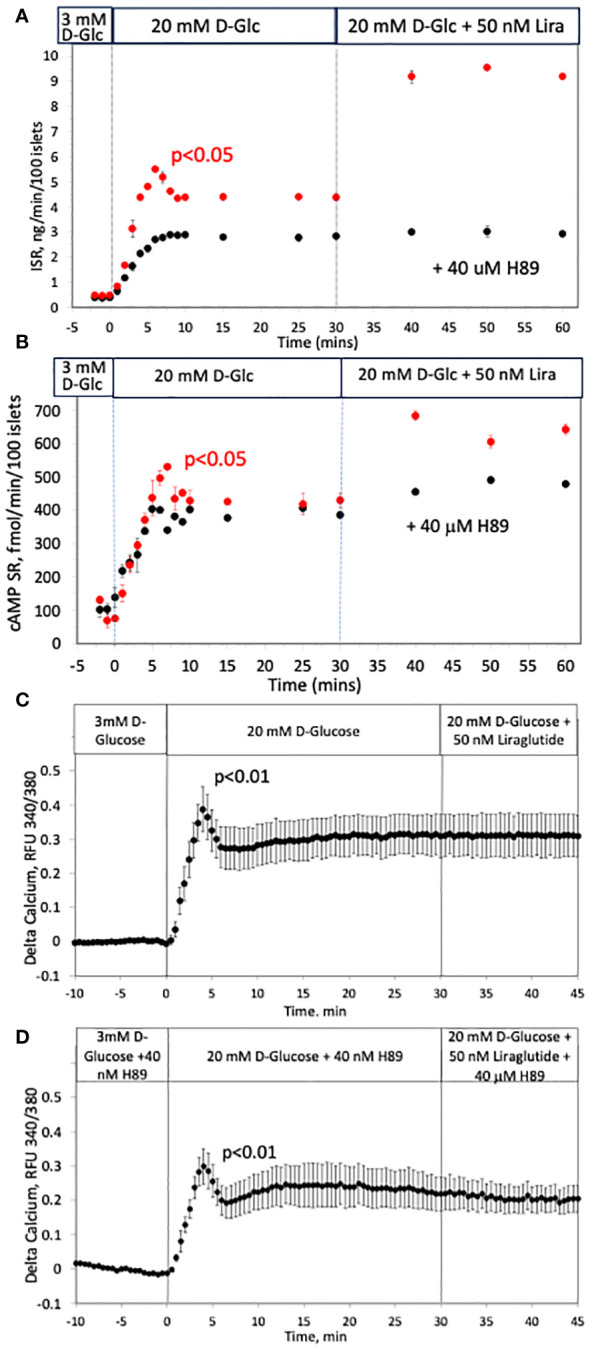
Effect of a blocker of kinases (H89) on glucose-stimulated ISR, cAMP SR and calcium in rat islets. **(A, B)** Insulin and cAMP SR were measured in fraction collected before and after the indicated increases in D-glucose and an agonist of GLP-1 receptor (liraglutide) in the absence (red circles, n = 2) and presence of a blocker of PKA (H89, black circles, n = 3); 50 islets/channel were perifused. Each data point is an average +/- SE. Flow rate was 250 mL/min in each 140 mL chamber. **(C, D)** The effect of a rapid rise in D-glucose and liraglutide on calcium in the absence **(C)** or presence **(D)** of H89. Each data point is an average +/- SE of 3 experiments where each one measured a single islet. Flow rates were 200 mL/min in each 100 mL chamber.

Having found that cAMP is released in a biphasic pattern, we strove to test whether the known effect of cAMP on ISR contributes to first-phase ISR. Exposure to a protein kinase inhibitor, H89, resulted in complete suppression of the first-phase portion of glucose-stimulated ISR (**Figure 8A**). The second phase, characterized by gradual increase in ISR which reached steady state after about 10 minutes, was about 30% lower for islets exposed to H89 than for control islets. Exposure to H89 largely blocked the effect of a glucagon-like peptide-1 receptor (GLP-1R) agonist (liraglutide) on ISR, consistent with the view that H89 inhibits PKA. However, as H89 is non-specific and acts on many kinases, we measured cAMP SR in response to H89. Somewhat unexpectedly, first-phase cAMP SR response, and response to liraglutide were nearly completely abolished by exposure to H89, while the levels at 12 mM D-glucose were unchanged (**Figure 8B**). It appears that cAMP SR is stimulated by H89-sensitive and H89-insensitive processes. It is notable that when glucose-stimulated calcium was measured in the presence and absence of H89 (**Figures 8C, D**), the shape and magnitude of the first-phase response of calcium in response to glucose was unaffected by H89. There was however, a diminished second-phase response of calcium in the presence of H89, consistent with inhibition of kinases leading to decrease overall cytosolic calcium levels. Despite reports that calcium can effect cAMP levels (69), no effect of the GLP-1R agonist liraglutide on calcium was observed, consistent with studies reporting that calcium and cAMP are not strongly temporally linked (70).

### Effect of osmotic pressure on first-phase ISR by mouse and human islets

**Figure 9:**
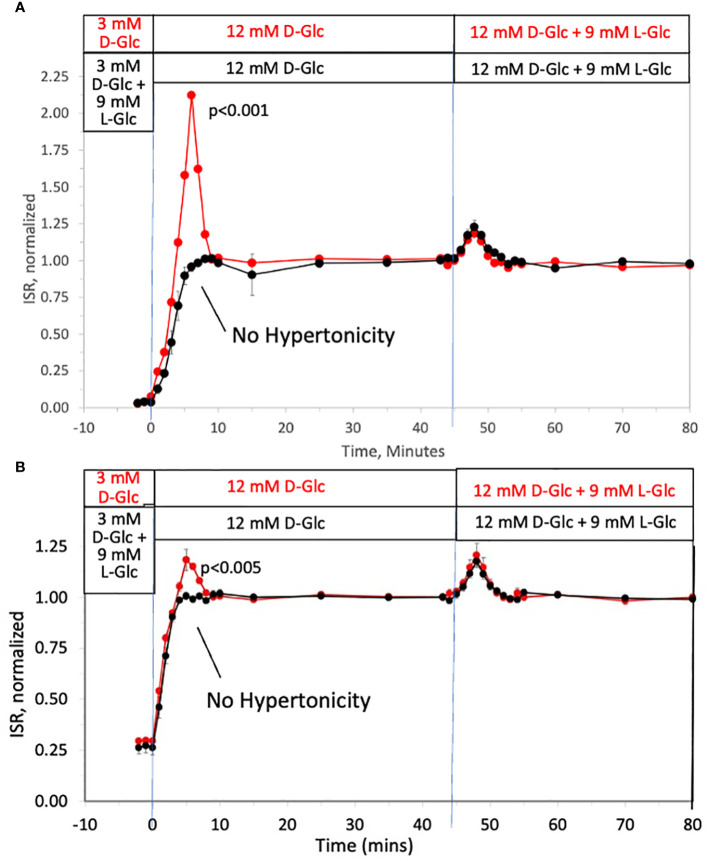
Dissection of osmotic vs metabolic components of biphasic insulin secretion in response to 12 mM D-glucose by mouse and human islets. ISR was measured in experiments just as described in **Figure 2A**. Each data point is an average +/- SE of 3 **(A)** or 7 **(B)** perifusions normalized to the steady-state value at 12 mM glucose calculated by dividing each value by the average ISR values between times of 25–45 minutes (A: 4.1 +/- 0.01 ng/min/100 islets for D-glucose alone and 4.0 +/- 0.04 ng/min/100 islets preceded by L-glucose; and B: 6.7 +/- 0.8 ng/min/100 islets for D-glucose alone and 6.3 +/- 0.8 ng/min/100 islets preceded by L-glucose). Flow rate was 250 mL/min in each 140 mL chamber.

To demonstrate that tonicity is mediating first-phase insulin secretion by islets of other species, mouse and human islets were tested for their response to L-glucose as was done for rat islets. The waveforms of first- and second-phase secretory responses of mouse islets have been reported to be somewhat different than by rat islets (71–73). In agreement with these reports, when performing the protocol performed for **Figure 2** on mouse islets, the first-phase of ISR was roughly three times the size of rat islets (**Figure 9A**), however, we found that the shapes and magnitudes of second-phase ISR were very similar. The response by human islets to increased D-glucose was also biphasic (**Figure 9B**), and consistent with studies with large numbers of human islet preparations (55), the magnitude of the first-phase was slightly smaller than for rat islets, while the second phase was similar in magnitude. Despite the variation in relative sizes of first-phase for mouse and human vs. rat islets, first-phase ISR by all islet types was not generated in response to D-glucose when changes in tonicity were prevented (**Figures 2A**, **9A, B**). As with rat islets, there were also transient responses of ISR to L-glucose by mouse and human islets, although for mouse islets it was smaller than the first-phase. The delay in the peak of first-phase ISR when D-glucose was rapidly increased vs. the peak when L-glucose elicited first-phase ISR after prior elevation of D-glucose was also consistent between the three islet species. Taken together, it appears that first-phase ISR in response to hypertonicity is an intrinsic property of the mechanisms mediating glucose-stimulated insulin secretion in rat, mouse, and human islets.

## Discussion

### Effects of hypertonicity on insulin secretion

It has been over 50 years since the discovery that islets secrete insulin in a biphasic manner in response to a rapid rise in glucose. During this time, a range of biochemical and biophysical mechanisms mediating the effect have been proposed (as reviewed (8–13)). The data in this paper strongly supports a novel mechanism mediating first-phase ISR that emanates from hypertonicity resulting from a rapid change in D-glucose which drives first-phase ISR. The direct approach of using cell impermeable carbohydrates to dissect out the osmotic forces from the effects of glucose metabolism was definitive in revealing the role of hypertonicity on stimulating first-phase responses of NAD(P)H, calcium, cAMP SR as well as ISR. Our data demonstrates osmotic effects on islet function that arise with changes in tonicity of as little as 3 mM L-glucose, well below the concentration that can induce detectable changes in volume. It has been suggested that KCl (in the presence of diazoxide) demonstrates the operation of triggering of first-phase secretion (74), where cytosolic calcium drives exocytosis (75), while increased metabolism drives the sustained, second phase of secretion (76). If we dissect out first and second phases out according to the stimulation of ISR by metabolism vs membrane-impermeable effects, the peak of the first-phase ISR did not occur until 3–4 minutes after the effects of D-glucose had begun. Thus, the current data challenges not only the mechanisms mediating first-phase ISR, but also the interrelationship where it appears that first-phase appears to be an amplification of second phase, the details of which will be discussed below.

### Intracellular signals mediating first phase ISR: calcium and cAMP

After generating clear evidence that hypertonic effects of cell impermeable carbohydrates mediate first-phase ISR, the molecular mechanisms that are mediating increased ISR were investigated in more detail. The two parameters known to most strongly mediate ISR, calcium and cAMP, were also both secreted in biphasic fashion in response to a rapid rise in D-glucose. Moreover, they were both also transiently elevated in a waveform similar to first-phase ISR in response to L-glucose in the presence of elevated D-glucose, raising the possibility that one or both signals is mediating the effect of hypertonicity on first-phase ISR. Evidence that first-phase calcium influx is driving the first-phase ISR is supported by the dependency of the transient increase in ISR on LTCCs, which in the presence of nimodipine was abolished. LTCCs are known to be responsible for facilitating the rise in cytosolic calcium that is preferentially linked to ISR (60–62, 77, 78), That both phases of ISR are dependent on LTCCs may indicate that the two phases converge on a single pathway, and it could be that biphasic ISR in response to glucose simply tracks calcium influx from LTCCs as has been previously suggested (9, 59, 78).

However, three observations argue against calcium as being the direct determinant of first-phase kinetics. The first is the delay in the peak of first-phase ISR in response to a rapid rise D-glucose concentration vs. when exposed to a rise in L-glucose in the presence of already elevated D-glucose. The second piece of evidence is the dependency of the magnitude of first-phase ISR in response to L-glucose on the prevailing concentrations of D-glucose. Taken together, these two pieces of evidence suggests that the effect of increasing ISR by hypertonicity is an amplifying factor that works when metabolism of D-glucose has already activated LTCCs. This interpretation is consistent with a third piece of evidence where blocking kinases with H89 abolished first-phase ISR while leaving first-phase calcium response intact. Thus, to the extent that first-phase calcium is driving first-phase ISR, it is likely doing this through stimulation of a calcium- and H89-sensitive kinase that amplifies glucose-stimulated ISR.

We then considered whether the first-phase effects of cAMP might be driving first-phase ISR as its action on PKA is well known to amplify ISR only when D-glucose is elevated and LTCCs are activated (79). The presence of an inhibitor of a protein kinases, H89, completely abolished first-phase ISR in response to D-glucose and largely abolished the response to a GLP1R agonist (liraglutide). Surprisingly, H89 also blocked first-phase but not second-phase response to glucose. The stimulation of cAMP SR by liraglutide – like ISR -, was also nearly abolished, removing PKA’s contribution to H89’s inhibitory effects on ISR as a factor. Irrespective, it appears that first-phase ISR in response to D-glucose may be mediated by hypertonicity that transiently amplifies glucose-stimulated ISR by an H89-sensitive process. It seems plausible that given 1. H89’s suppression of cAMP/PKA-stimulated ISR, 2. a first-phase pattern of cAMP SR similar to that of first-phase ISR, and 3. that cAMP/PKA are well-established to potentiate glucose-stimulated ISR, cAMP stimulation of PKA may be an important mediator of hypertonic-driven first-phase ISR. However, this interpretation is tempered by the lack of specificity of H89, which is capable of inhibiting a large number of kinases (80). Indeed, it has been reported that H89 inhibits more than 25 proteins including CAMK and AMPK (81). Therefore, more work has to be done to definitively identify what kinases are mediating the H89-sensitive process(es), in part by testing other kinase inhibitors. Thus, it is possible that first-phase calcium response, despite its insensitivity to H89, could be stimulating a kinase capable of amplifying ISR which is inhibitable by H89. For instance, CAMK was a target and is both calcium-sensitive and its inhibition has been shown to reduce ISR (48, 82).

### Use of cAMP SR as a measure of cAMP regulation

The regulation of ISR by cAMP has been studied extensively (see review (64)). The two most common methods to measure cAMP are the measurement of total cAMP content of islets, and more recently imaging of intracellular cAMP by FRET imaging analysis. Neither of these methods provide the resolution of both the kinetics and magnitude of cAMP responses needed to evaluate the subtle and transient changes that occurred in response to a rapid response to glucose. The measurement of total cAMP after lysing islets does yield absolute values in terms of mass/islet, However, the dynamic range of total cAMP content is low and effects of glucose on cAMP content are not always observed (83, 84), probably due to much of the cAMP being bound (85). Importantly, the temporal resolution of lysing islets precludes it as a method to evaluate rapid transient responses. The use of FRET methodologies to measure cAMP does improve the kinetic resolution, and real time changes in response to GLP-1 and IBMX are revealed (86). Nonetheless, the changes from these methods are fluorescent signals that are not readily converted into actual cAMP concentrations and the dynamic range of changes was only 30–60% (86, 87). Indeed, using these two methods, kinetic responses of cAMP in response to an increase in glucose did not resemble those of biphasic insulin secretion observed in our study (70, 88).

To resolve the kinetics and the magnitude of the cell signal cAMP, the secretion rate of cAMP into the extracellular outflow from the perifusion system was calculated similarly to the calculation of ISR. In this way, the resolution of kinetics of the two parameters are comparable, and the magnitude of response in release are accurately measured by a simple ELISA assay on fractions obtained without the perturbing the islets. A feature of the method does indeed yield a much larger dynamic range of responses than either of the other methods as a change in D-glucose from 3 to 12 mM elicited a more than 300% increase in cAMP SR, a value more than 3–10 times what was seen with either total content (89) or FRET (87). However, the approach does not measure intracellular levels of cAMP, and our interpretations above were based on the supposition that the cAMP SR is proportional to the intracellular levels. Given that changes in cAMP SR occurred rapidly in response to D-glucose, this may be true. However, it could also be that cAMP is exported out of the cell under regulation that is not related to its intracellular concentration, and possibly within insulin secretory granules. Under the latter condition, cAMP SR would be expected to correlate with ISR even without a causal relation to first-phase ISR. Despite these caveats, the causal relationship between cAMP and ISR, suggests that the increase in cAMP SR seen in response to D- and L-glucose is consistent with a contribution of cAMP to a transient amplification of ISR that drives first-phase ISR.

### Causes of a short duration of first-phase ISR

The short and transient waveform of the first-phase secretory response which lasts only a few minutes is consistent with the fact that glucose transport into beta cells is fast (90), and the relaxation of the disequilibrium between intracellular and extracellular glucose will depend on the relative rate of transport versus metabolism of glucose (91). However, the fact that L-glucose (and mannitol) also elicited a transitory secretory response suggests that the transient nature of the effect is not due solely to dissipation of difference in glucose concentration across the plasma membrane, but compensation by intrinsic mechanisms that function to re-equilibrate electrical/ionic gradients, water transport and cell volume back to pre-hypertonic conditions. The lack of transport of the membrane impermeable compounds might be expected to elicit a larger hypertonicity-driven spike in ISR than D-glucose, but this was not seen, further indicating that cellular processes dissipate the effects of hypertonicity that were largely independent of magnitude of change in tonicity.

### Stretch-activated channels proposed to mediate effects of glucose on cell swelling

A mechanism mediating the response of islets to glucose-induced changes in osmotic pressure that has received a fair amount of attention is through a class of stretch-activated cation channels that include piezo1, Tentonin 3/TMEM150, TRPV2 and LRRC8/VRAC (42–45). The concept is based on build-up of metabolic intermediates from glucose such as lactate leading to hypotonic-induced entry of water into islet cells that results in cell swelling (38). The resultant expansion of the plasma membranes during cell swelling activates stretch-activate cation channels, and inhibition of these proteins result in a diminution of glucose-stimulated ISR. Given that the effects seen in our study were in response to membrane-impermeable compounds that do not enter cells, it seems that mechanisms mediating the transient effects observed in this study do not involve stretch-activated channels. Furthermore, hypertonicity, not hypotonicity must be eliciting the responses, consistent with a requirement for a rapid increase in glucose to cause first-phase kinetics.

### Mechanisms mediating effects of hypertonicity

A major question that remains to be answered is how hypertonicity elevates NAD(P)H, calcium and cAMP. It has been observed in studies of liver that hypertonicity enhances glycolysis as reflected by an increase in lactate/pyruvate production from glucose (92), however, no mechanisms were elucidated/described to explain this effect. In general, when exposed to fuels (such as glucose or KIC), the resulting increase in NAD(P)H reflects increased ATP generation leading to opening of LTCCs (35). Thus, it is plausible that increased metabolism is a primary event as a result of hypertonicity, which leads to increases in cAMP and calcium. If this is the case though, it is not clear why the increase in NAD(P)H at low D-glucose would not increase calcium and cAMP, unless at sub-threshold glucose metabolic rate is not increase sufficiently by hypertonicity to elicit changes in cAMP or calcium.

The most studied mechanisms leading to changes in osmotic pressure involve fluxes of ions such as sodium, potassium and chloride (93, 94), which are strongly linked to metabolic rate (95–97). Cell volume regulation is very complex and involves multiple ion channels and pumps, water transport, metabolic pathways (possibly via changes in volume of mitochondrial matrix (98)). Given the small change in tonicity that was needed (3 mM L-glucose) to elicit a transient change in ISR, it doesn’t seem likely that this would induce enough of a volume change to be significant. A report describing a 5% cell swelling in response to 16 mM of the non-metabolizable glucose analog 3-O-methylglucose, while no change in membrane potential was observed (38). argues against either of these processes mediating hypertonicity-induced changes. However, there is also supporting evidence that changes in hypertonicity are linked to depolarization of plasma membranes (92, 99) which seems more likely than volume to mediate effects and is consistent with the increased intracellular calcium that we observed in response to glucose. As we only did experiments on whole islets, we also cannot ascertain what cell types in the islets generated cAMP or contributed to the NAD(P)H and calcium signals.

### Summary: a proposed simple model for first-phase ISR

**Figure 10:**
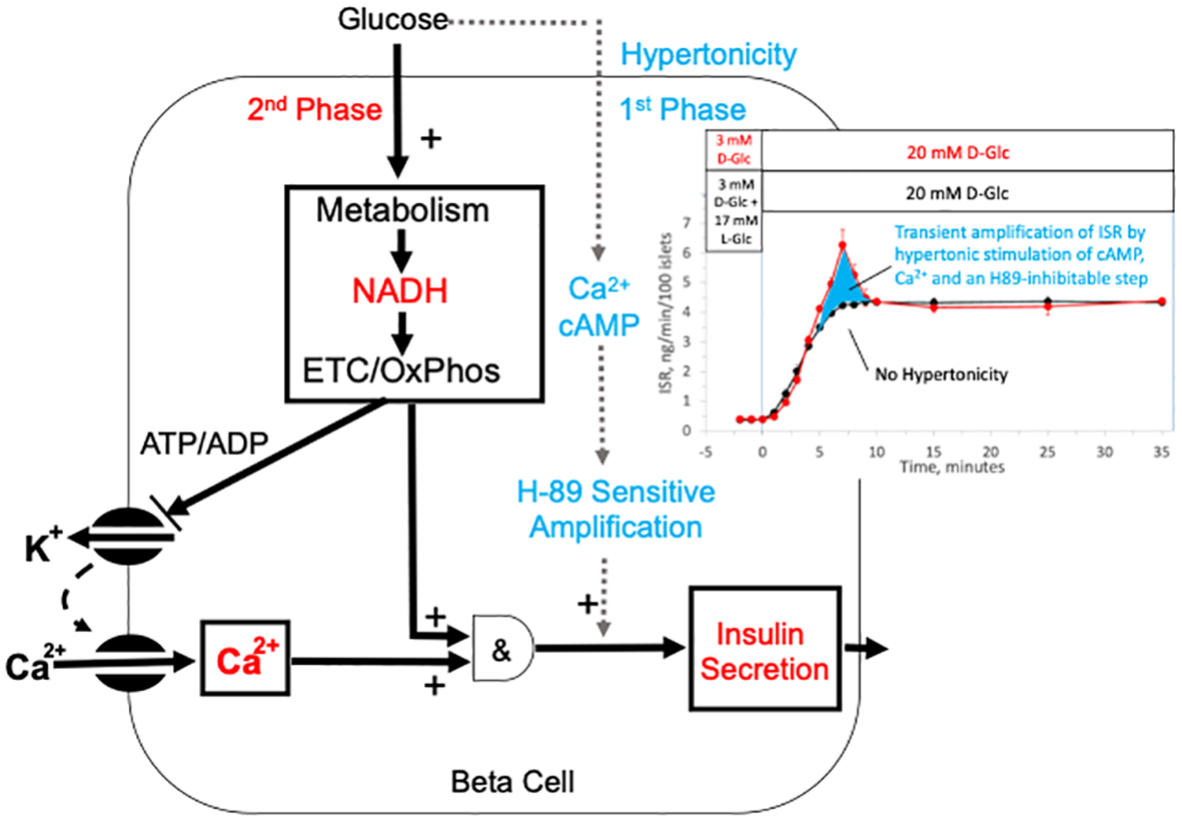
Model of mechanisms mediating glucose-induced biphasic ISR. The schematic depicts well established mechanisms for driving second-phase ISR including the metabolism of glucose, closing of KATP channels, activation of LTCCs, as well as the metabolic generation of factors that with calcium activate ISR. Our data supports a novel mechanism for first-phase ISR that is driven by a rapid but transient change in hypertonicity leading to transient spikes in cAMP, calcium and ISR, where the first-phase ISR is mediated by an amplification of the mechanisms mediating 2nd phase insulin by cAMP and/or stimulation of an H89-sensitive kinase (possibly PKA). The hypertonicity stemming from a rapid rise in glucose is likely short-lived as intrinsic islet responses to the increased osmotic pressure operate to restabilize ionic gradients and cell volume. The inset shows the dissection of first- and second-phase ISR (from **Figure 1A**) as the difference between the effects of a rapid change in D-glucose with and without a change in tonicity.

As enumerated in the Introduction, there are many mechanisms that have been proposed to account for first-phase ISR involving biophysical and biochemical mechanisms, some of which involve proteins that specifically respond to changes in tonicity. However, with the data collected for this study, we are not in a position to support or rule out the simultaneous or mediative operation of their involvement. Nevertheless, hypertonicity is unique as an explanation of transient first-phase response to agents that are impermeable to the cell membrane, and it also explains the dependency on the speed of glucose ramp for the generation of first-phase insulin secretion. The observations that like ISR, major components known to mediate second-phase ISR (calcium and cAMP) both displayed hypertonic-stimulated first-phase responses that were also dependent on elevated glucose and activation of LTCCs suggest a confluence of hypertonicity induced signals with the well-accepted consensus model describing sustained ISR. Based on the findings that first-phase ISR is blocked by H89, a non-specific kinase inhibitor that blocked cAMP SR and liraglutide’s effects on glucose-stimulated ISR, while calcium responses were unchanged. we favor a dominant role for cAMP or calcium to stimulate a H89-senstive kinase that transiently amplifies ISR. The timing in upslopes of ISR in response to D-glucose whether in the presence or absence of hypertonicity were similar, demonstrating the lack of contribution from hypertonic-stimulated first-phase on this early response to D-glucose. Although we did not set out to test the fundamental concept that first-phase insulin secretion (referred to as triggering) precedes second-phase (100), our data challenges this view. The data suggests that hypertonically-stimulated first-phase ISR is not occurring prior to activation of mechanisms mediating second-phase ISR. The findings of our study can be summarized with a very simple model where first-phase ISR is simply hypertonic stimulation of an H89-sensitive protein kinase that transiently amplifies second-phase ISR possibly driven by cAMP and/or calcium (as depicted in **Figure 10**).

Our unique experimental approach allowed clear dissection of the waveforms of two phases and although we realize there could be alternate explanations, this simple model fits both the delayed response of first-phase relative to the sustained (second) phase of ISR glucose response as well as the glucose concentration dependency imparted by the glucose-dependency of amplification effects. The revelation that first-phase responses by islets to rapidly increasing glucose involves steps mediated by the osmotic pressure is an important piece of the puzzle, and if true, would have a large impact on interpretation of first-phase data in basic and clinical research on islet function and diabetes research. Given that loss of first-phase ISR is commonly observed in individuals with Type 2 diabetes, and that patients with long standing diabetes have been subjected to repeated and chronic hypertonic excursions, this current study should stimulate more efforts to understand mechanisms mediating the beta cell’s response to hypertonicity.

## Methods

### Chemicals

Krebs-Ringer Bicarbonate buffer was used for all perifusions prepared as described previously (46). Antimycin A, D-glucose, KCN, nimodipine, mannitol, L-glucose and H-89 were all purchased from Sigma-Aldrich, and liraglutide was purchased from Selleck Chemicals (Houston, TX). Gas cylinders containing 21% O_2_/5% CO_2_ and balance N_2_ were purchased from Praxair Distribution Inc (Danbury CT).

### Islet preparation and procurement

*Sex as a biological variable.* Our study exclusively examined islets harvested from male rats, mice and humans. It is unknown whether the findings are relevant for female mice, but here is no evidence that islets from females have differences in the fundamental mechanisms mediating glucose-stimulated insulin secretion.

R*at islet isolation and culture.* Islets were harvested and pooled from typically 3 male Sprague-Dawley rats (approximately 250 g; Envigo/Harlan, Indianapolis, IN) anesthetized by intraperitoneal injection of sodium pentobarbital (150 mg/kg rat)) and purified as described (101, 102). All procedures were approved by the University of Washington Institutional Animal Care and Use Committee. Subsequently, islets were cultured for 18 hours in RPMI Media 1640 supplemented with 10% heat-inactivated fetal bovine serum (Invitrogen) at 37°C prior to the experiments.

*Mouse islets isolation and culture.* The study was approved by the Institutional Animal Care and Use Committee of the VA Puget Sound Health Care System. Mouse islets were isolated from pancreata of 7- to 8-week-old male C57BL/6.NEP+/+ mice (that were bred and housed in a colony maintained at the VA PSHCS and the secretory characteristics of their islets have been described (103)) by collagenase digestion as previously described (104). Briefly, pancreata were digested by intraductal injection of 3mL Collagenase P (0.5 mg/mL) in RPMI 1640 (with L-glutamine) containing 100 U/mL penicillin, 100 lg/mL streptomycin, and 11.1mM glucose. Following incubation at 37 degrees C for 15 min, pancreatic tissue was disrupted by shaking by hand for 1 min. Islets were then purified using a Histopaque-1077 density gradient. After islets were freed from exocrine tissue, they were hand-picked under a stereoscopic microscope (Olympus, Tokyo, Japan) and transferred for overnight culture in RPMI 1640 medium with 10% (vol/vol) heat-inactivated fetal calf serum, in a 37°C humidified atmosphere of 95% air:5% CO_2_.

*Human islet procurement and culture.* Human islets from cadaveric donors were obtained from the Integrated Islet Distribution Program. Islet donor and isolation characteristics, including donor age, sex, BMI, cause of death, measurements of islet purity and viability, ischemia duration, and culture time, are provided in **Supplementary Table 1**. Upon receipt, islets were incubated overnight in RPMI 1640 under the same conditions that were used for rat and mouse islets prior to perifusion analysis.

### Perifusion system for the measurement of ISR and cAMP SR response

The perifusion system used in this study was one that we have used extensively in the past and have previously described in detail (46–48). Briefly, when measuring ISR and cAMP SR, islets (50 or 25/channel as indicated in the figure legends) were loaded into vertical, glass perifusion columns (volume = 140 μL) and onto frits that resided about 1/4^th^ of the way from the inflow of the tube). The inflow to the columns (typically 8 done in parallel) was supplied by a peristaltic pump that drew perifusate from various bottles containing the indicated composition, and the outflow from each column was collected in a multi-channel fraction collector. To stimulate first-phase responses, the flow rates were higher than we have typically used in the past. Although it is likely that first-phase responses might have been larger at faster flow rates, flow rates of 250 uL/min were used for all ISR experiments to minimize islet movement during experiments. Prior to each experiment, islets cultured overnight in RPMI1640 were washed with KRB containing 3 mM glucose (and 0.1% BSA), and pre-equilibrated in a CO_2_ incubator at 37°C. To fully ensure that islets had reached a steady state at 3 mM glucose KRB, all perifusion experiments began with a 90-minute baseline period before measuring responses to test compounds. Data for individual channels from multiple perifusions and islet isolations were combined. Note that oscillations in ISR would not be visible due to the transit time for flow to reach the fraction collector and the low sampling rate.

### Assays for glucose, insulin, and cAMP

To determine the rate of change in glucose that contact islets in the perifusion system, glucose was measured in outflow fractions collected during experiments using a kit (Cat no. A22189, Invitrogen, Carlsbad, CA) and following the manufacturer’s instructions. To measure rates of insulin secretion and efflux of cAMP, outflow fractions were assayed by RIA (Cat no. RI-13K, Millipore Sigma, Burlington, MA) or ELISA (Rodent Insulin Chemiluminescence ELISA, Catalog 80-INSMR-CH10, Alpco, Salem, NH) for insulin and competitive chemiluminescent immunoassay (cAMP-Screen Direct™ Cyclic AMP Immunoassay System, Catalog number: 4412186, Applied Biosystems, Foster City, CA) for cAMP. Amounts of insulin and cAMP in inflow samples were insignificant, so rates of secretion/production were calculated as the concentration in the outflow times the flow rate and normalized by the number of islets.

### Imaging and quantification of cytosolic NAD(P)H and Ca^2+^

NAD(P)H and cytosolic Ca^2+^ were both measured by fluorescence imaging of single isolated islets, where NAD(P)H is detected as islet autofluorescence with 360 nm excitation and 460 nm emission (which reflects the sum of NADH and NAD(P)H (105)), and calcium was measured after loading islets with Fura-2 AM (Invitrogen) as previously described (48). For these imaging experiments, the same perifusion system that was used for ISR and cAMP SR was used, but the vertical perifusion column was replaced with a temperature-controlled, 100-μl perifusion dish (Bioptechs, Butler, PA) that was mounted on to the stage of a Nikon Eclipse TE-200 inverted microscope (48). Flow rates of 200 uL/min were used for all imaging experiments, lower than that used for the vertical perifusion column (which has a 20% higher chamber volume) in order to match the rate of change in glucose in the two systems.

Results for calcium are displayed as the ratio of the fluorescent intensities during excitation at two wavelengths (F340/F380) after subtracting off the baseline acquired at 3 mM D-glucose. To normalize NAD(P)H data, at the end of the protocol KCN was added (to determine the maximum NAD(P)H signal) followed by exposure to FCCP (which reduces the NAH(P)H signal to background levels). The normalized fluorescence data was then calculated as follows (**Equation 1**),


(1) $$\%\text{ Reduced NAD}\left(\text{P}\right)\text{H }=100\text{ x }\left(\text{RF}{\text{U}}_{\text{test}}–\text{ RF}{\text{U}}_{\text{FCCP}}\right)/\left(\text{RF}{\text{U}}_{\text{KCN}}–\text{ RF}{\text{U}}_{\text{FCCP}}\right)$$

where RFU_FCCP_ and RFU_KCN_ equaled the averages of the final 10 time points each agent was present.

### Statistical analysis

Data was plotted as the average +/- the Standard Error (SE), where SE was calculated as Standard Deviation/n^1/2^. Statistical significance was determined using Student’s t tests carried out with Microsoft Excel (Redmond, WA) in two ways. The first was to compare the statistical significance of the transient peaks with and without counterbalancing the change in tonicity when D-glucose is rapidly increased. The second is to determine whether the transient is significant by performing t-tests comparing the peak to the steady state values. The selection of statistical analyses is indicated on the Figure legends. Since this study involved studying waveforms where interpretations were not dependent on absolute values, in some statistical analysis and graphing, data was normalized to the steady state values at 20 mM glucose. When this was done, the steady-state values used to normalize the data are reported in the figure legends.

For glucose concentration dependencies, data was fit to a Hill Plot represented as (**Equation 2**)


(2) $$\text{ISR }=\text{ IS}{\text{R}}_{\text{max}}\text{x }{\left[\text{Glucose}\right]}^{\text{n}}/\left({\left[\text{Glucose}\right]}^{\text{n}}+{\left({\text{S}}_{0.5}\right)}^{\text{n}}\right)$$

where ISR_max_ is the maximal rate of ISR, S_0.5_ is the concentration at which ISR is half maximal in mM, and *n* is the Hill coefficient which governs the sigmoidicity of the concentration dependency.

## Data availability statement

The original contributions presented in the study are included in the article/**Supplementary Material**. Further inquiries can be directed to the corresponding author.

## Ethics statement

The studies involving humans were approved by Human Subjects Division, University of Washington. The studies were conducted in accordance with the local legislation and institutional requirements. The human samples used in this study were acquired from Human pancreatic islets and were provided by the NIDDK-funded Integrated Islet and Distribution Program (IIDP) at City of Hope, (2UC4DK098085). Written informed consent for participation was not required from the participants or the participants’ legal guardians/next of kin in accordance with the national legislation and institutional requirements. All procedures were approved by the University of Washington Institutional Animal Care and Use Committee. and the Institutional Animal Care and Use Committee of the VA Puget Sound Health Care System. The study was conducted in accordance with the local legislation and institutional requirements.

## Author contributions

VK: Data curation, Methodology, Writing – review & editing. IS: Conceptualization, Funding acquisition, Investigation, Methodology, Supervision, Validation, Writing – original draft, Writing – review & editing, Project administration, Resources.
