# Supplementary material for: Hypertonicity during a rapid rise in D-glucose mediates first-phase insulin secretion
Source: Front Endocrinol (Lausanne). 2024 Jun 26;15:1395028. doi: 10.3389/fendo.2024.1395028 (PMC11233695; doi:10.3389/fendo.2024.1395028)
Supplement: Supplementary file 2 [file DataSheet_1.pdf]

Islet Preparation	6/8/22	12/7/23
**Mandatory Information**		
Unique Identifier	SAMN28867622	SAMN38606565
Donor Age (yrs)	36	47
Donor Sex (M/F)	M	M
Donor BMI (kg/m^2)	29.6	26.3
Donor HbA1c	5.4	5.5
Origin/Source of islets	IIDP	IIDP
Islet isolation center	Southern California Islet Cell Resource Center, City of Hope	Southern California Islet Cell Resource Center, City of Hope
Donor history of diabetes (yes/no)?	No	No
**Recommended Information**		
Cause of Death	Anoxia	Cerebrovascular/Stroke
Warm ischemia time (h)	0.42	0
Cold ischemia time (h)	8.18	11.57
Estimated purity (%)	93	80
Estimated viability (%)	96	95
Total culture time (h)	45	44
Data/time islet culture began at islet isolation center	6/4/22 12:05	12/2/23 6:00
Handpicked to purity? Yes/No	Yes	Yes
